# Foetal loss after chorionic villus sampling and amniocentesis in twin pregnancies: A multicentre retrospective cohort study

**DOI:** 10.1002/pd.6237

**Published:** 2022-09-27

**Authors:** Kate Navaratnam, Delima Khairudin, Robyn Chilton, Andrew Sharp, George Attilakos, Daniel Stott, Sophie Relph, Rebecca Spencer, Dominique A. Badr, Andrew Carlin, Jacques Jani, Mark D. Kilby, Mercede Sebghati, Asma Khalil, Zarko Alfirevic

**Affiliations:** ^1^ Fetal Medicine Unit Liverpool Women’s Hospital Liverpool UK; ^2^ Harris‐Wellbeing Research Centre University of Liverpool Liverpool UK; ^3^ Fetal Medicine Unit University College London Hospital NHS Foundation Trust and Institute for Women's Health University College London London UK; ^4^ University of Leeds and Fetal Medicine Unit Leeds Teaching Hospitals NHS Trust Leeds UK; ^5^ Department of Obstetrics and Gynecology University Hospital Brugmann Université Libre de Bruxelles Brussels Belgium; ^6^ Institute of Metabolism & Systems Research College of Medical & Dental Sciences University of Birmingham Birmingham UK; ^7^ Fetal Medicine Centre Birmingham Women's and Children's Foundation Trust Birmingham UK; ^8^ Fetal Medicine Unit St George's Hospital St George's University of London London UK

## Abstract

**Objective:**

We aimed to determine foetal losses for DCDA and MCDA twins following transabdominal CVS or amniocentesis performed <22+^0^ weeks.

**Methods:**

Retrospective cohort study conducted in the UK and Belgium 01/01/00–01/06/20. Cases with unknown chorionicity, monochorionic complications or complex procedures were excluded. Uncomplicated DCDA and MCDA twins without invasive procedures were identified as controls. We reported foetal losses <24+^0^ weeks and losses of genetically and structurally normal foetuses.

**Results:**

Outcomes were compared for DCDA foetuses; 258 after CVS with 3406 controls, 406 after amniocentesis with 3390 controls plus MCDA foetuses, 98 after CVS with 1124 controls, and 160 after amniocentesis with 1122 controls. There were more losses <24+^0^ weeks with both procedures in DCDA (CVS RR 5.54 95% CI 3.38–9.08, amniocentesis RR 2.36 95% CI 1.22–4.56) and MCDA twins (CVS RR 5.14 95% CI 2.51–10.54, amniocentesis RR 7.01 95% CI 3.86–12.74). Losses of normal foetuses were comparable to controls (DCDA CVS RR 0.39 95% CI 0.05–2.83, DCDA amniocentesis RR 1.16 95% CI 0.42–3.22, MCDA CVS RR 2.3 95% CI 0.71–7.56, and MCDA amniocentesis RR 1.93 95% CI 0.59–6.38).

**Conclusions:**

This study indicates increased foetal losses for DCDA and MCDA twins following CVS and amniocentesis with uncertain risk to normal foetuses.

## Key points

What's already known about this topic?

- Monochorionic twins are at a higher baseline risk of foetal loss, but outcome data for these pregnancies following CVS and amniocentesis are limited.
- Procedure‐related losses following CVS and amniocentesis in twins overall may be lower than previously reported.
- Procedures themselves may not contribute significantly to pregnancy loss for women at a higher baseline risk but may be more influential for women at lower a priori risk.

What does this study add?

- Spontaneous losses of twins prior to 24 weeks are increased after CVS and amniocentesis for both DCDA and MCDA pregnancies compared to controls.
- Losses of structurally and genetically normal twins appear comparable to control groups. However, due to low numbers of adverse outcomes in our cohort and other published analyses, further evidence is required.
- Maternal counselling should highlight different baseline risks between MCDA and DCDA twins and the contribution of maternal and pregnancy factors to outcomes for complex twin pregnancies.

## 1 INTRODUCTION

Despite advances in non‐invasive testing, chorionic villus sampling (CVS) and amniocentesis are the prenatal tests of choice for diagnosing chromosomal and genetic abnormalities. Assessment in a randomised controlled trial (RCT) is the gold standard to estimate a procedure‐related risk. However, it is unlikely that appropriately powered, high‐quality randomised controlled trials, evaluating comparative effects of CVS and amniocentesis would be feasible or acceptable to patient groups.

A recent meta‐analysis reported a weighted pooled procedure‐related risk of miscarriage for singletons of 0.35% for both CVS and amniocentesis.^1^ Accepting the limitations of observational data and relative imprecision of the estimated risks, recent RCOG guidance concluded that procedure‐related risk of miscarriage for singleton pregnancy is likely to be less than 0.5%.^2^, ^3^

Evidence quantifying procedure‐related risks in multiple pregnancies is significantly more limited. Observational studies published in the last 2 decades suggest that the procedure‐related risk of miscarriage in twins may be lower than previous estimates.^4^ A systematic review published in 2020 included 2713 amniocentesis procedures and 349 CVS in multiple pregnancies. The results indicated no significant difference in foetal loss before 24 weeks gestation and within 4 weeks of procedure, compared to those not undergoing an invasive procedure. These findings are further supported by a recent large multicentre retrospective cohort study that used logistic regression. The authors found no significant contribution of CVS to the risk of post‐procedure miscarriage. Interestingly, when the same dataset was analysed using propensity scoring, there was a 3.5% higher absolute risk of foetal loss following CVS for women at a low baseline risk.^5^, ^6^ Therefore, more evidence is needed to establish a robust evidence base and clarify inconsistent study findings.

Attempts to quantify procedure‐related risks in multiple pregnancies are challenging. Published case series tend to be small with variation in definitions of procedure‐related foetal loss.^4^ Monochorionicity, with shared vascular connections, confers additional complexity.^7^ Increased baseline risks, technical challenges, and post‐procedure losses may be attributable to the monochorionicity rather than the procedure itself and yet this information is often unavailable or inadequately reported.

To improve the clinical utility of the available data, we decided to analyse dichorionic diamniotic (DCDA) and monochorionic diamniotic (MCDA) twin pregnancies as two distinct cohorts from the outset. For each group, we sought to identify structural and genetically normal foetal losses and look for phenotypic differences in types of foetal loss.

## 2 METHODS

This retrospective, multicentre cohort study included women with DCDA and MCDA twin pregnancies managed between 01/01/00 and 01/06/20 in six tertiary foetal medicine centres in the UK and Belgium, namely Birmingham Women's and Children's Foundation NHS Trust (BWH), Leeds Teaching Hospitals NHS Trust, Liverpool Women's Hospital NHS Foundation Trust (LWH), St George's Hospital, University College London Hospital and University Hospital Brugmann (UHB), Brussels.

Routinely collected clinical data were sought and therefore ethical approval was not required, according to the Health Research Authority decision tool.^8^ Audit approval was obtained in each centre. All women were identified via electronic searches of local databases and medical records.

The control groups comprised women managed and delivering in LWH or UHB between 01/01/09 and 01/06/20 with two viable foetuses on scan between 11+^0^ and 14+^0^ weeks. These two centres were chosen for representative comparator groups for the study population overall. Both centres have comprehensive electronic records covering the chosen time period to allow identification of uncomplicated twin pregnancies.

The most appropriate control groups would comprise twins that had an indication for testing but declined prenatal diagnosis. However, as sufficiently large numbers would not be identifiable, we chose to compare with uncomplicated twin pregnancies that had not undergone invasive diagnostic or therapeutic procedures. Pregnancies where chorionicity could not be determined or unobtainable outcomes were excluded. MCMA pregnancies, those with TRAP, TTTS, or discordant structural or genetic abnormalities were excluded from control groups. Pregnancies affected by foetal loss between 11+ 0 and 15+^0^ weeks were excluded from the amniocentesis control group.

Women who had undergone CVS (11+^0^–22+^0^ weeks) or amniocentesis (15+^0^–22+^0^ weeks) in six centres between 01/01/00 and 01/06/20 were considered as the study group. Exclusion criteria included pregnancies where chorionicity could not be determined or with unobtainable outcomes. MCMA pregnancies, those with TRAP, and TTTS were excluded. We also excluded pregnancies when more than two attempts were required to obtain a sample, when procedures were performed alongside embryo reduction or when selective foetocide was performed within the reporting period (<24+^0^ weeks).

Twin chorionicity and amnionicity were determined by ultrasound scan in the first trimester as per evidence‐based guidance.^9^, ^10^ CVS and amniocentesis were carried out by foetal medicine specialists, using continuous ultrasound guidance and aseptic technique. 17–21G needles were used for CVS, 20–22G for amniocentesis. All CVS procedures were performed transabdominally and intra‐amniotic dye infusion was not used for amniocentesis. Foetal wellbeing was assessed upon procedure completion.

For controls, CVS, and amniocentesis groups, we chose to assess losses at 24^+0^ weeks as, below this threshold, twin foetuses are unlikely to be viable. For controls, CVS, and amniocentesis groups, we reported overall foetal losses <24+^0^ weeks, including deaths of both foetuses and single foetal deaths and any reported deaths of structurally and genetically normal foetuses <24+^0^ weeks. For CVS and amniocentesis groups, we also reported overall foetal losses <24+^0^ weeks within 15 days of procedure, including death of both foetuses and single foetal deaths. We also reported any deaths of structurally and genetically normal foetuses within 2 weeks (<15 days) of the procedure as more likely to be related to the procedure.

Maternal demographic data were identified including indication for testing. Technical details of procedures and their complications were identified, including sampling from one or both twins, use of the same or separate needle (single puncture or separate punctures), bloodstaining of sample, maternal cell contamination, and failure to obtain a result. Anatomical findings, genetic results, and outcomes for both twins at 24+^0^ weeks gestation (number of foetuses alive) were extracted. For tertiary referrals, pregnancy outcome data were sought from referring hospitals via standard email request and followed up by telephone.

Statistical analyses were undertaken using SPSS version 27 (IBM Corp. Released 2020; IBM SPSS Statistics for Window, Armonk, NY: IBM Corp). Demographic and pregnancy data were reported per pregnancy; outcomes were reported per foetus. Relative risk (RR) with 95% confidence intervals (CI) was calculated for all outcomes following CVS and amniocentesis in monochorionic and dichorionic twins versus controls. Computational issues may occur when no events are observed in one or both groups analysed. Where no events occurred, a recognised zero cell correction was applied by adding 0.5 to affected cells prior to the analysis.^11^

Forest plots to compare our data to those recently published were generated in Revman 5.4 (The Cochrane Collaboration, available at revman.cochrane.org). Risk ratios with random effects were calculated for CVS and amniocentesis in DCDA and MCDA twins, respectively. Where judged appropriate, data were pooled.^2^

## 3 RESULTS

**FIGURE 1:**
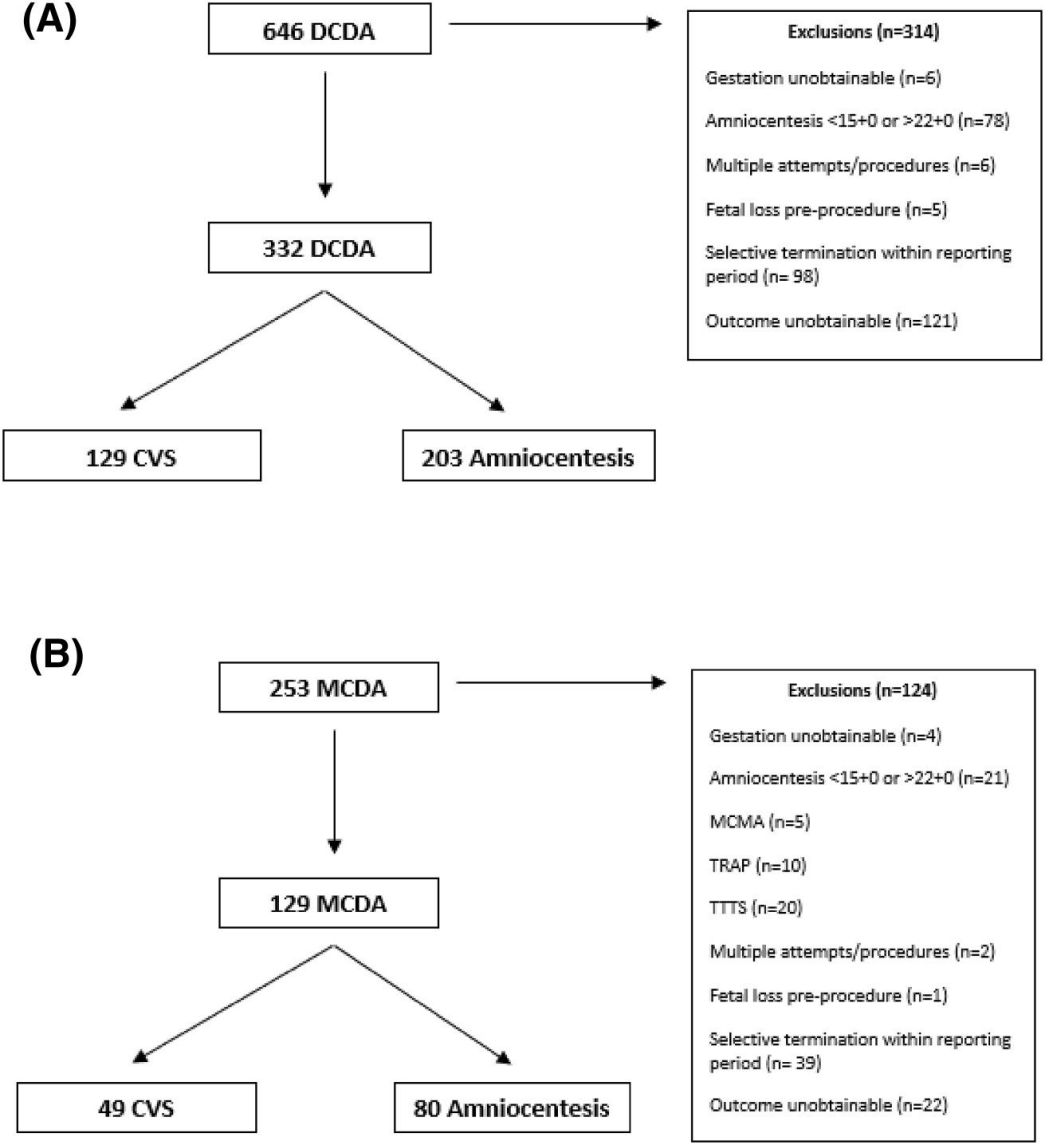
Study flow diagram for (A) dichorionic diamniotic twin pregnancies (DC) and (B) monochorionic diamniotic twin pregnancies undergoing CVS or amniocentesis

Changes in patient record systems in BWH and UHB meant that data for women who had invasive procedures could only be obtained between 01/01/06 and 30/09/19 in BWH and between 01/01/09 and 01/06/20 in UHB. In total, 899 women with twin pregnancies had CVS or amniocentesis during the study period. After exclusions, 129 women with DCDA twins had CVS and 203 had an amniocentesis. After exclusions, 49 women with MCDA twins had CVS and 80 had amniocentesis (Figure 1).

There were 2284 twin pregnancies that had not undergone invasive procedures. Seven DCDA and 12 MCDA cases were excluded, leaving 1703 DCDA pregnancies (3406 foetuses) and 562 MCDA pregnancies (1124 foetuses) as the control group for CVS. Eight DCDA and one MCDA cases affected by foetal losses between 11 + 0 and 15+^0^ were excluded, leaving 1695 pregnancies (3390 foetuses) and 561 pregnancies (1122 foetuses) as the control group for amniocentesis.

The median maternal age for DCDA controls was 32 years (IQR 28–36), whereas it was 35 years (IQR 31–39) for those who had prenatal diagnosis. The median age of MCDA controls was 30 years (interquartile range, IQR 26–34), whereas it was 34 years (IQR 29–38) for those who had prenatal diagnosis. Indications for prenatal diagnosis are shown in Table 1.

**TABLE 1:** Indications for prenatal diagnosis in dichorionic diamniotic and monochorionic diamniotic twin pregnancies

Indication	DCDA twin pregnancies (*n* = 332)		MCDA twin pregnancies (*n* = 129)	
	CVS (%)	Amniocentesis (%)	CVS (%)	Amniocentesis (%)
Aneuploidy screen positive	87 (67.4)	102 (50.2)	23 (46.9)	37 (46.2)
Structural anomaly	22 (17.0)	59 (29.1)	13 (26.5)	29 (36.2)
Foetal growth restriction	5 (3.9)	8 (3.9)	2 (4.1)	6 (7.5)
Previous affected pregnancy or chance of inherited condition	10 (7.7)	15 (7.4)	9 (18.4)	5 (6.2)
Maternal request	1 (0.8)	17 (8.4)	1 (2.0)	2 (2.5)
Others	2 (1.5)	0 (0)	0 (0)	1 (1.2)
Not stated	2 (1.5)	2 (1.0)	1 (2.0)	0 (0)

## 4 DICHORIONIC DIAMNIOTIC TWINS (DCDA)

In the CVS control group, 1.5% (50/3406) of foetuses died before 24+^0^ weeks, comprising 17 twin pair deaths (1%) and 16 single foetal deaths (0.5%). In the amniocentesis control group, 1.2% (39/3390) of foetuses died before 24 + 0 weeks, comprising 0.8% (28/3390) as twin pairs and 0.3% (11/3390) single foetal deaths (Table 2a).

**TABLE 2:** Outcomes for (2a) DCDA twins following CVS or amniocentesis and control DCDA twins; (2b) MCDA twins following CVS or amniocentesis and for control MCDA twins

(a)				

Outcome		CVS		Amniocentesis
	Controls	*n* = 258	Controls	*n* = 406
	*n* = 3406	RR (95% CI)	*n* = 3390	RR (95% CI)
Total twins alive at 24^+0^ weeks	3356 (98.5%)	237 (91.9%)	3351 (98.8%)	395 (97.3%)
		0.93 (0.90–0.97)		0.98 (0.97–1.00)
Both twins in pair alive at 24^+0^ weeks	3340 (98.1%)	220 (85.3%)	3340 (98.5%)	388 (95.6%)
		0.88 (0.83–0.92)		0.97 (0.95–0.99)
Total twins lost <24^+0^ weeks	50 (1.5%)	21 (8.1%)	39 (1.2%)	11 (2.7%)
		5.54 (3.38–9.08)		2.36 (1.22–4.56)
Single twin demise	16 (0.5%)	17 (6.6%)	11 (0.3%)	7 (1.7%)
		14.03 (7.17–27.43)		5.31 (2.07–13.63)
Both twins demised	34 (1.0%)	4 (1.6%)	28 (0.8%)	4 (1.0%)
		1.55 (0.56–4.34)		1.19 (0.42–3.38)
Losses of normal twins^a^	50 (1.5%)	0 (0%)^b^	39 (1.2%)	4 (1.3%)
		0.39 (0.05–2.83)		1.16 (0.42–3.22)

(b)				

Outcome		CVS		Amniocentesis
	Controls	*n* = 98	Controls	*n* = 160
	*n* = 1124	RR (95% CI)	*n* = 1122	RR (95% CI)
Total twins alive at 24^+0^ weeks	1102 (98.0%)	86 (87.8%)	1102 (98.2%)	140 (87.5%)
		0.90 (0.83–0.96)		0.89 (0.84–0.95)
Both twins in pair alive at 24^+0^ weeks	1086 (96.6%)	84 (85.7%)	1086 (96.8%)	136 (85.0%)
		0.89 (0.82–0.96)		0.88 (0.82–0.94)
Total twins lost <24^+0^ weeks	22 (2.0%)	10 (10.2%)	20 (1.8%)	20 (12.5%)
		5.14 (2.51–10.54)		7.01 (3.86–12.74)
Single twin demise	16 (1.4%)	4 (4.1%)	16 (1.4%)	4 (2.5%)
		2.8 (0.98–8.41)		1.75 (0.59–5.18)
Both twins demised	6 (0.5%)	6 (6.1%)	4 (0.4%)	16 (10%)
		11.47 (3.77–34.89)		28.05 (9.50–82.85)
Losses of normal twins^a^	22 (2.0%)	3 (4.5%)	20 (1.8%)	3 (3.4%)
		2.3 (0.71–7.56)		1.93 (0.59–6.38)

^a^ Losses of normal twins comprise genetically and structurally normal twins. Losses in this group are shown as a percentage of all genetically and structurally normal twins.

^b^ Zero cell correction applied for analysis.^11^

### 4.1 CVS

In 62.3% (81/129) DCDA CVS, both placentas were sampled, and in 27.2% (22/81), separate needle insertions were used. All samples were obtained at the first attempt.

Following CVS in DCDA twin pregnancies, there was a significant increase in total foetal deaths prior to 24+^0^ weeks (8.1%, 21/258, RR 5.54 95% CI 3.38–9.08), mostly due to single foetal deaths (6.6%, 17/258, RR 14.03 95% CI 7.17–27.43) (Table 2a).

Eleven foetuses died within 2 weeks of CVS, including two twin pairs, and seven foetuses survived by their co‐twin (Table S1a).

There were no losses of structurally and genetically normal foetuses in the CVS group, which were comparable to losses that occurred in controls (1.5%, 50/3406, RR 0.39 95% CI 0.05–2.83) (Table 2a).

### 4.2 Amniocentesis

In 80.3% (163/203) DCDA amniocentesis, both amniotic sacs were sampled, and in 87.7% (143/163), separate needle insertions were used. Three cases required a second attempt to obtain the sample.

Following amniocentesis in DCDA twins, there was also a significant increase in total foetal deaths prior to 24+^0^ weeks (2.7%, 11/406, RR 1.85 95% CI 0.97–3.52), also largely composed of single foetal deaths (1.7%, 7/406, RR 3.67 95% CI 1.52–8.87) (Table 2a).

Three foetuses died within 2 weeks of amniocentesis, including one twin pair and one foetus survived by their co‐twin (Table S1b).

Overall, there were two losses of pairs of genetically and structurally normal twins, including the twin pair that died within 2 weeks of amniocentesis (Table S1b). Similar losses of normal foetuses were observed in the amniocentesis group and control group (1.3% 4/300 vs. 1.2% 39/3390 RR 1.16 95% CI 0.42–3.22) (Table 2a).

## 5 MONOCHORIONIC DIAMNIOTIC TWINS (MCDA)

In the CVS control group, 2.0% (22/1124) of foetuses died before 24+^0^ weeks, comprising 0.5% (6/1124) as twin pairs and 1.4% (16/1124) single foetal deaths. In the amniocentesis control group, 1.8% (20/1122) of foetuses died before 24 + 0, comprising 0.4% (4/1122) within twin pairs and 1.4% (16/1122) single foetal deaths (Table 2b).

### 5.1 CVS

In 34.7% (17/49) MCDA CVS, both placentas were sampled, and in 17.6% (3/17), separate needle insertions were used. One case required a second attempt to obtain the sample.

Following CVS in MCDA twin pregnancies, there was a significant increase in total foetal deaths prior to 24+^0^ weeks (10.2%, 10/98, RR 5.14 2.51–10.54), with a significant increase in deaths of twin pairs (6.1%, 6/98, 3.77–34.89) (Table 2b).

Seven foetuses demised within 2 weeks of CVS including, three twin pairs and one foetus that was survived by their co‐twin (Table S2a).

Overall, there were three losses of structurally and genetically normal foetuses, none of which occurred within 2 weeks of CVS (Table S2a). More losses of normal foetuses were observed in the CVS group compared to controls, but with wide confidence intervals crossing the null value due to small overall numbers (4.5% 3/66 vs. 22/1124 RR 2.3 95% CI 0.71–7.56) (Table 2b).

### 5.2 Amniocentesis

In 65% (52/80) MCDA amniocentesis, both amniotic sacs were sampled, and in 96.2% (50/52), separate needle insertions were used. Two cases required a second attempt to obtain the sample.

Following amniocentesis, there was a significant increase in total foetal deaths prior to 24+^0^ weeks (12.5%, 20/160, RR 6.39 95% CI 3.57–11.43) with a significant increase deaths of twin pairs (10%, 16/160, RR 18.73 95% CI 7.44–47.17) (Table 2b).

Fifteen foetuses died within 2 weeks of the procedure, one that was survived by their co‐twin and seven twin pairs (Table S2b).

There were three losses of genetically and structurally normal foetuses alongside an affected co‐twin, two of which occurred within 2 weeks of amniocentesis (Table S2b).

Losses of normal foetuses were comparable in the amniocentesis and control groups with wide confidence intervals crossing the null value due to small overall numbers (3.4% 3/87 vs. 1.8% 20/1122 RR 1.93 95% CI 0.59–6.38) (Table 2b).

## 6 DISCUSSION

### 6.1 Main findings

This retrospective cohort study demonstrates that spontaneous losses of twins prior to 24 weeks are increased after CVS and amniocentesis for both DCDA and MCDA pregnancies compared to controls. Whilst losses of structurally and genetically normal twins appear comparable to control groups, the confidence intervals are very wide, and therefore, the data must be interpreted cautiously. Our data are compatible with no difference in risk of normal twin demise between cases and controls, but also with clinically significant differences in both directions. Whilst a clinically important increase in risk cannot be ruled out, there is a possibility that the risk is actually decreased for those undergoing prenatal diagnosis.

### 6.2 Strengths and limitations

Our multicentre retrospective cohort study includes a relatively large number of DCDA and MCDA twin pregnancies and reflects the provision of contemporary specialised prenatal diagnosis for complicated twin pregnancies with advances in ultrasound technology and invasive techniques. Strict exclusion criteria were applied to ensure that twin subpopulations with higher intrinsic risks of foetal loss did not impact overall outcomes, including MCMA pregnancies, and those affected by TRAP and TTTS. Importantly, we have reported outcomes by chorionicity, described procedural factors, and provided detailed relevant foetal information for all post‐procedure demises (Supplemental Tables S1 and S2).

An important limitation of our dataset is considerable demographic data loss due to under‐reporting of maternal BMI, ethnicity, and parity, which is a limitation in describing a priori risk of loss. This was largely due to the inclusion of tertiary referral cases and changes in patient information systems during the study period. Despite efforts made to follow‐up outcome data from referring units, some data were not recoverable. It is possible that further adverse outcomes were contained within these missing data. Missing data were more prevalent for DCDA pregnancies as they were more likely to be discharged back to local care. It has recently been shown that although parity does not influence foetal loss, higher maternal weight and black racial origin do contribute to increase the risk.^5^

Our strict exclusion criteria led to relatively small numbers of eligible cases. Changes to patient records meant that older cases could not be reliably searched in two centres. Whilst we made every effort to include appropriate control groups, we do not have post‐mortem information for all losses in the control groups. Without this information, we cannot exclude the possibility of losses due to undiagnosed TTTS.

Heterogeneity of technique for CVS and variable reporting of techniques for both procedures are potential confounders for our study and others. We collected all available information on techniques and samples (Tables S1a and S1b, S2a and S2b). However, monochorionic CVS may be carried out using a single sampling site or by sampling adjacent to each placental cord insertion to guard against unidentified heterokaryotypic monozygotism.^12^ Similar limitations have affected all other published cohorts.

### 6.3 Interpretation

The best evidence regarding procedure‐related risks for CVS and amniocentesis will continue to come from observational data. The accumulated evidence in singleton pregnancies indicates that CVS and amniocentesis pose a reassuringly small additional risk of pregnancy loss.^13^ The limiting factors for such estimates in twin pregnancies are relatively low numbers of procedures and very low event rates. Data are especially limited for monochorionic twin pregnancies. A recent systematic review of singletons included 13 times the number of procedures that were included in a similar systematic review in twins.^13^, ^14^

Monozygosity and monochorionicity confer additional risks that mark these twin pregnancies as a distinct population.^7^ Our data indicate a similar risk of post‐procedure demise for CVS and amniocentesis in MCDA twins; however, these risks are appreciably higher than those we observed in DCDA twins (Tables 2a and b). This observation supports our assertion that dichorionic or pooled twin outcomes should not be extrapolated to the monochorionic population for the purposes of evidence‐based guidance and patient counselling.

Since the most recent systematic review of procedure‐related loss in twin pregnancies, two further observational datasets have been published.^5^, ^15^ Dechnunthapiphat et al. reported post‐amniocentesis demises prior to 24 weeks, but did not stratify outcomes by chorionicity.^15^ Data from eight foetal medicine units in the UK, Spain, Italy, Bulgaria, and Portugal were analysed in two separate papers. Using logistic regression Elger et al. (2021) concluded that losses prior to 24 weeks gestation increased two‐fold following CVS, but the procedure itself did not contribute significantly to the risk of foetal loss.^5^ The authors attributed the increase in foetal losses to maternal and pregnancy factors.^5^ When, in an attempt to deal with any potential confounding of maternal and pregnancy factors, the same dataset was analysed using propensity score matching, Gil et al. reported a significant (3.5%) increase in the individual risk following CVS for women at the low baseline risk.

### 6.4 Clinical and research implications

Our findings of increased post‐procedure losses following CVS and amniocentesis in both DCDA and MCDA twin pregnancies and possible clinically important risks to normal twins stand in contrast to the conclusions of recent published studies.

It is important to stress that data derived from small studies with significant heterogeneity will always limit precision of summary statistics. Even when data are sufficiently homogeneous for clinically meaningful pooled analysis, lack of statistical significance does not exclude a possibility of clinically important differences. This is particularly relevant when dealing with low event rates causing bias towards statistical non‐significance. When the 95% confidence interval includes 1, the ‘logical’ conclusion is that there is no difference in procedure‐related loss. It should be noted, however, that our data are compatible not only with an increase in procedure‐related loss but also with the possibility of a clinically significant ‘protective’ effect from invasive procedures. Whilst this could be ignored as implausible, it is not inconceivable that women with MCDA twins undergoing prenatal diagnosis may relax after testing and rest from work. They may benefit from enhanced care with expert imaging and increased surveillance. We encourage future authors not to focus solely on the statistical significance, but to interpret the data much more holistically.

## 7 CONCLUSION

This study indicates increased twin losses prior to 24 weeks following CVS and amniocentesis in MCDA and DCDA twin pregnancies and these findings contrast somewhat with recently published evidence. The uncertain procedure‐related risk to structurally and genetically normal twins persists due to low numbers of adverse outcomes in our cohort and other published analyses. Any counselling should highlight different baseline risks between MCDA and DCDA twins and the contribution of maternal and pregnancy factors to outcomes for complex twin pregnancies.

## CONFLICT OF INTEREST

The authors declare that there is no conflict of interest that could be perceived as prejudicing the impartiality of the research reported.

## Supporting information

Supplementary Material 1

Supplementary Material 2

Supplementary Material 3

## Data Availability

Data available in article supplementary material.
