# Supplementary material for: Foetal loss after chorionic villus sampling and amniocentesis in twin pregnancies: A multicentre retrospective cohort study
Source: Prenat Diagn. 2022 Sep 27;42(12):1554–61. doi: 10.1002/pd.6237 (PMC9828484; doi:10.1002/pd.6237)
Supplement: Supplementary file 1 — Supplementary Material 1 [file PD-42-1554-s001.docx]

**Table S1.** DCDA twin losses following CVS **(S1a)** and amniocentesis **(S1b)**. *Indicates loss of two structurally and chromosomally normal twins.

| **Number of DC twins lost** | **Timing of loss after CVS** | **Procedure Factors** | **Structural/functional issues (1 or 2 twins)** | **Genetic results**  **(1 or 2 twins)** |
| --- | --- | --- | --- | --- |
| 1 | ≤ 14 days | Both placentas sampled, single needle insertion | Anencephaly (1) | Normal (2) |
| 1 | ≤ 14 days | Both placentas sampled separate needle insertions | Normal (2) | Trisomy 21 (1)  Normal (1) |
| 1 | ≤ 14 days | Both placentas sampled, single needle insertion | CRL <1st centile (1) | Normal (2) |
| 1 | ≤ 14 days | Both placentas sampled, single needle insertion | Exomphalos (1) | Trisomy 18 (1)  Normal (1) |
| 1 | ≤ 14 days | Both placentas sampled, single needle insertion | DORV (1) | Normal (2) |
| 1 | ≤ 14 days | Both placentas sampled, single needle insertion | NT >3.5mm (1) | Trisomy 21 (1)  Normal (1) |
| 2 | ≤ 14 days | Both placentas sampled, separate needle insertions | Normal (2) | Trisomy 21 (2) |
| 2 | ≤ 14 days | Both placentas sampled, separate needle insertions | Normal (2) | Trisomy 21 (2) |
| 1 | ≤ 14 days | Both placentas sampled, separate needle insertions | Normal (2) | Trisomy 18 (1)  Normal (1) |
| 1 | >14 days | Both placentas sampled, separate needle insertions | Normal (2) | Trisomy 18 (1), mosaic trisomy 18 (1) |
| 1 | >14 days | Both placentas sampled separate needle insertions | Exomphalos (1) | 48XXX, mosaic trisomy 8 (1) |
| 1 | >14 days | Single placenta sampled, maternal cell contamination | Cystic Hygroma (1) | Failed result, MCC (1) |
| 1 | >14 days | Both placentas sampled separate needle insertions | Normal (2) | Normal (2) |
| 1 | >14 days | Single placenta sampled | Hydrops Fetalis (1) | Normal (1) |
| 1 | >14 days | Both placentas sampled separate needle insertions | NT >3.5mm (1) | 45X (1) |
| 1 | >14 days | Both placentas sampled, single needle insertion | NT >3.5mm (1) | Trisomy 21 (1) |
| 1 | >14 days | Both placentas sampled, single needle insertion | Holoprosencephaly (1) | Normal (2) |
| 1 | >14 days | Single placenta sampled | NT >3.5mm (1) | Trisomy 18 (1) |
| 1 | >14 days | Single placenta sampled | NT >3.5mm (1) | Normal (1) |

| **Number of DC twins lost** | **Timing of loss after Amnio** | **Procedure Factors** | **Structural/functional issues (1 or 2 twins)** | **Genetic results**  **(1 or 2 twins)** |
| --- | --- | --- | --- | --- |
| 1 | ≤14 days | Both sacs sampled, separate needle insertions | Holoprosencephaly (1) | Trisomy 13 (1)  Normal (1) |
| 1 | ≤14 days | Both sacs sampled, separate needle insertions | Bilateral hydrothorax (1) | Trisomy 21 (1)  Normal (1) |
| 1 | ≤14 days | Both sacs sampled, separate needle insertions | SGA, VSD (1) | Trisomy 21 (1)  Normal (1) |
| 1 | ≤14 days | Both sacs sampled, separate needle insertions | NT >3.5mm, AVSD (1) | Trisomy 21 (1)  Normal (1) |
| 1 | ≤14 days | Both sacs sampled, separate needle insertions | NT >3.5mm (1) | Trisomy 21 (1)  Normal (1) |
| 1 | ≤14 days | Both sacs sampled, separate needle insertions | NT >3.5mm (1) | Trisomy 13 (1)  Normal (1) |
| 1 | ≤14 days | Both sacs sampled, separate needle insertions | Normal (2) | Complex chromosomal rearrangement (1)  Normal (1) |
| 2* | ≤14 days | Both sacs sampled, single needle insertion | Normal (2) | Normal (2) |
| 2* | >14 days | Both sacs sampled, separate needle insertions | Normal (2) | Normal (2) |
