# Supplementary material for: Foetal loss after chorionic villus sampling and amniocentesis in twin pregnancies: A multicentre retrospective cohort study
Source: Prenat Diagn. 2022 Sep 27;42(12):1554–61. doi: 10.1002/pd.6237 (PMC9828484; doi:10.1002/pd.6237)
Supplement: Supplementary file 2 — Supplementary Material 2 [file PD-42-1554-s002.docx]

**Table S2.** MCDA twin losses following CVS **(S2a)** and amniocentesis **(S2b)**. * Indicates pregnancy loss that included a genetically and structurally normal co-twin.

| **Number of MC twins lost** | **Timing of loss after CVS** | **Procedure Factors** | **Structural/functional issues**  **(1 or 2 twins)** | **Genetic results**  **(1 or 2 twins)** |
| --- | --- | --- | --- | --- |
| 1 | ≤14 days | Single placenta sampled | Megacystis (1) | Normal (1)  Unknown (1) |
| 1 | ≤14 days | Single placenta sampled | Raised NT (2) | Normal (1)  Unknown (1) |
| 1 | ≤ 14 days | Both placentas sampled | NT >3.5mm (2) | 45X (2) |
| 1 | ≤ 14 days | Both placentas sampled | Hydrocephalus (1) | Normal (2) |
| 2* | >14 days | Both placentas sampled | sFGR (1) | Normal (2) |
| 2* | >14 days | Single placenta sampled | Raised NT (1) | Normal (1)  Unknown (1) |
| 2* | >14 days | Single placenta sampled | Multiple anomalies (1) | Normal (1)  Unknown (1) |

| **Number of MC twins lost** | **Timing of loss after Amnio** | **Procedure Factors** | **Structural/functional issues**  **(1 or 2 twins)** | **Genetic results**  **(1 or 2 twins)** |
| --- | --- | --- | --- | --- |
| 2 | ≤14 days | second attempt, both amniotic sacs sampled with separate needle insertions | Hydrops Fetalis (2) | Trisomy 21 (2) |
| 1 | ≤14 days | both amniotic sacs sampled with separate needle insertions | Normal (2) | Trisomy 21 (1)  Normal (1) |
| 1 | ≤14 days | both amniotic sacs sampled with separate needle insertions | Bilateral hydrothorax (1) | Normal (2) |
| 2* | ≤14 days | both amniotic sacs sampled with separate needle insertions | sFGR | Normal (2) |
| 2 | ≤14 days | both amniotic sacs sampled with separate needle insertions | Cystic Hygroma, VSD (1) | 45X (1)  Normal (1) |
| 2 | ≤14 days | both amniotic sacs sampled with separate needle insertions | Normal (2) | Trisomy 21 (2) |
| 2 | ≤14 days | both amniotic sacs sampled with separate needle insertions | NT >3.5mm (2) | Trisomy 21 (2) |
| 2* | ≤14 days | both amniotic sacs sampled with separate needle insertions | Univentricular heart, hydrops fetalis (1) | Normal (2) |
| 2* | ≤14 days | both amniotic sacs sampled with separate needle insertions | Cardiac anomaly, hydrops fetalis (1) | Normal (2) |
| 2 | ≤14 days | both amniotic sacs sampled with separate needle insertions | NT >3.5mm (2) | DNA stored, not tested (2) |
| 1 | ≤14 days | both amniotic sacs sampled with separate needle insertions | Structural anomaly (1) | Normal (2) |
| 1 | >14 days | both amniotic sacs sampled with separate needle insertions | FGR, posterior fossa cyst, cardiac anomaly (1) | Normal (2) |
