# Supplementary material for: Foetal loss after chorionic villus sampling and amniocentesis in twin pregnancies: A multicentre retrospective cohort study
Source: Prenat Diagn. 2022 Sep 27;42(12):1554–61. doi: 10.1002/pd.6237 (PMC9828484; doi:10.1002/pd.6237)
Supplement: Supplementary file 3 — Supplementary Material 3 [file PD-42-1554-s003.docx]

**Table S3.** Contribution of each participating centre to final cohort

| **Centre** | **CVS** | | **Amniocentesis** | |
| --- | --- | --- | --- | --- |
|  | **MC** | **DC** | **MC** | **DC** |
| Liverpool | 6 | 15 | 23 | 59 |
| UCLH | 4 | 18 | 8 | 20 |
| Birmingham | 12 | 30 | 24 | 63 |
| Brugmann | 3 | 6 | 7 | 8 |
| St George’s | 14 | 49 | 10 | 18 |
| Leeds | 10 | 11 | 8 | 35 |
| **TOTAL** | **49** | **129** | **80** | **203** |
